# Biological, Molecular, and Physiological Characterization of Four Soybean Mosaic Virus Isolates Present in Argentine Soybean Crops

**DOI:** 10.3390/v17070995

**Published:** 2025-07-16

**Authors:** Mariel Maugeri, Marianela Rodríguez, Nicolas Bejerman, Irma G. Laguna, Patricia Rodríguez Pardina

**Affiliations:** 1 Instituto de Patología Vegetal IPAVE CIAP INTA, Av. 11 de Septiembre 4755, Córdoba X5020ICA, Argentina; mariel_maugeri@hotmail.com (M.M.); bejerman.nicolas@inta.gob.ar (N.B.); 2 Instituto de Fisiología y Recursos Genéticos Vegetales, Centro de Investigaciones Agropecuarias-INTA, Av. 11 de Septiembre 4755, Córdoba X5020ICA, Argentina; rodriguez.marianela@inta.gob.ar; 3 Unidad de Estudios Agropecuarios (UDEA-CONICET), Av. 11 de Septiembre 4755, Córdoba X5020ICA, Argentina; 4 Unidad de Fitopatología y Modelización Agrícola (UFyMA-CONICET), Av. 11 de Septiembre 4755, Córdoba X5020ICA, Argentina; irmagracielalaguna@yahoo.com.ar

**Keywords:** *Glycine max*, potyvirus, physiological alterations, *Potyvirus glycitessellati*, viral genome

## Abstract

Soybean mosaic virus (SMV) causes systemic infections in soybean plants, leading to chlorotic mosaic and significant yield losses. In Argentina, during the 1990s, three isolates were collected in Marcos Juárez (MJ), Manfredi (M), and Northwestern Argentina (NOA), along with the “Planta Vinosa” (PV) isolate, which causes severe necrosis in some cultivars. These isolates were freeze-dried and stored at −70 °C for several years. They were recovered by mechanical inoculation and biologically, molecularly, and physiologically characterized for the first time. Three of the four isolates showed low genetic divergence in the P1, CI, and CP genes. Although SMV-NOA and SMV-PV had high nucleotide sequence identity, they differed in pathogenicity, seed mottling, and transmission efficiency by seeds or aphids. SMV-NOA caused early changes in photosystem II quantum efficiency (ɸPSII) and malondialdehyde (MDA) content before symptom expression (BS). After symptom development (LS), SMV-M significantly increased MDA, total soluble sugars, and starch compared to the other isolates. Thus, early changes in ɸPSII and sugars may influence late viral symptoms. Likewise, SMV-MJ induced more severe symptoms in the susceptible Davis cultivar than in Don Mario 4800. Therefore, our results demonstrate genomic, biological, and physiological differences among SMV isolates and variable interactions of SMV-MJ with two soybean cultivars.

## 1. Introduction

Soybean [*Glycine max* (L) Merr.] is one of the most important legume crops and a source of edible oil and proteins. Argentina is the third world soybean producer, with 84% of the production being exported as grain, flour, oil, or biodiesel [1]. Extensive and intensive production of soybean with little genetic diversity is particularly vulnerable to attack by pathogens that can reduce yield and seed quality, and even devastate large cultivation areas. Virus diseases of soybean have become increasingly prevalent, affecting this crop worldwide. Soybean mosaic virus (SMV) is recognized as the most serious, long-standing problem in many soybean-producing areas in the world [2]. Infection by SMV usually causes yield losses typically ranging from 8% to 35%; however, losses of up to 94% have also been reported [3].

SMV induces variable symptoms, from small and sometimes almost unnoticeable chlorotic spots to large chlorotic areas. Other possible symptoms include mosaic, vein clearing, blistering, leaflet deformation, and internode shortening [4]. When plants are infected with severe virus strains, the virus can induce necrotic areas in petioles, stems, and leaves [5]. SMV also induces several types of seed mottling, with the most common one being “hilum bleeding”, caused by the spread of the hilum color across the seed coat. SMV-infected seeds can result in infected plants that serve as the initial inoculum, with later infections resulting from aphid transmission. Seed infection can be as high as 75%, depending on the soybean cultivar and the virus strain, but is usually less than 5% [6,7].

SMV is a member of the genus *Potyvirus*, in the *Potyviridae* family. It has a monopartite single-strand, positive-sense RNA genome that encodes a large polyprotein of about 350 kDa. This polyprotein is cleaved to yield at least 11 proteins: potyvirus 1 (P1), helper component proteinase (HC-Pro), potyvirus 3 (P3), P3/PIPO, a fusion product resulting from ribosomal frameshifting or transcriptional slippage at the conserved G1-2A6-7 motif near the 5′ end of pipo [8], 6kDa 1 (6K1), cylindrical inclusion (CI), 6 kDa 2 (6K2), nuclear inclusion a-viral protein genome linked (NIa-VPg), nuclear inclusion a-protease (NIa-Pro), nuclear inclusion b (NIb), and coat protein (CP) [9]. Several SMV isolates were classified into different strains based on their differential response in susceptible and resistant soybean cultivars [10,11,12,13,14]. Different types of responses of susceptible and resistant cultivars are the result of specific interactions between the soybean R gene product and the virus avirulence (Avr) gene product. At least three independent loci (*Rsv1*, *Rsv3*, and *Rsv4*) in the United States and several *Rsc* loci in China conferring resistance to different SMV strains have been reported [15,16]

Identifying SMV strains is crucial for both soybean cultivation and breeding efforts. Although pathogenicity-based methods have been widely used, they are labor-intensive and time-consuming. In recent years, genomic sequencing has emerged as an alternative approach to differentiate SMV strains. Among viral proteins, P1 is one of the most variable and informative proteins for strain comparison [17,18].

Studying the molecular variability and genetic structure of viruses helps provide an understanding of their molecular evolutionary history in relation to virulence, dispersion, and the emergence of new epidemics [19]. These studies focused mainly on phylogenetic relationships between virus isolates because most of the viruses are constantly evolving through genetic exchanges (recombination), as well as accumulation of mutations [20,21,22]. Due to the rapid evolution in avirulence/effector genes, the resistance conditioned by genes will be quickly overcome, and it is important to generate strategies for the management of viral diseases that are sustainable over time [15]. There is a significant demand to identify plant factors involved in defense responses to pathogens that can facilitate the design of new sustainable tolerance/resistance strategies against SMV. Therefore, it is necessary to know the impact of viruses on plant physiology, as well as the mechanisms and processes involved in the infection. In this context, previous studies identified the DAG motif in the coat protein (CP) as a major determinant of aphid transmission in SMV, with HC-Pro playing a secondary role. Seed transmission, in contrast, is influenced by the combined action of P1, HC-Pro, and CP [23,24]. Several motifs, including KITC and PTK, were detected in the HC-Pro protein that are involved in aphid transmission, as well as several amino acid residues (H256 in CP and R455 in HC-Pro), and G12 in the DAG motif, which are crucial in HC-CP interaction [25]. On the other hand, the resistance-gene-mediated defense response starts when pathogen effectors or the cofactors that bind to these effectors are detected by the host [26]. Seo et al. [27] found that the cylindrical inclusion (CI) protein acts as an elicitor of Rsv3-mediated extreme resistance and a pathogenic determinant, provoking lethal systemic hypersensitive response (LSHR) in Jinpumkong-2 cultivar. In addition, both P3 and HC-Pro are involved in the virulence of SMV on *Rsv1*-genotype soybean [28], and P3 protein is related to *Rsv4* resistance [29].

A compatible plant–virus interaction causes deleterious systemic effects on plants because viruses have the ability to reprogram the plant metabolism to their own benefit [30,31]. Virus infections that produce chlorotic symptoms affect photosynthetic capacity and carbohydrate metabolism and accelerate the leaf senescence process [30,31]. Reprogramming of metabolism includes suppression of plant defense responses, reallocation of photoassimilates, and redox imbalance, reduced photosynthesis, and induced senescence [30,32].

Five SMV isolates were initially characterized in Argentina: G1, G5, G6 (USA), and MS1 and MS2 (Brazil). In addition, during the 1990s, three geographic isolates of this virus—Marcos Juárez (MJ), Manfredi (M), and Northwestern Argentina (NOA)—along with an isolate known as “Planta Vinosa” (PV), which causes severe necrotic symptoms in certain cultivars, were collected for further characterization. The aim of the present study was to carry out the biological, physiological, and molecular characterization of these four isolates. Accordingly, this work provides, for the first time, integrated information on the genetic and physiological alterations involved in the SMV–soybean interaction. Studying historical SMV isolates is essential for understanding the evolution of viral pathogenicity, monitoring changes in virulence and host interactions over time, and developing effective long-term management strategies for soybean crops.

## 2. Materials and Methods

### 2.1. Inoculum Source

Plants with SMV symptoms were collected from four soybean-production areas of three provinces of Argentina: Marcos Juárez (MJ) and Manfredi (M) from Córdoba province, Salta (NOA), and Venado Tuerto, Santa Fe (Figure S1). The isolate identified in Santa Fe causes severe necrotic symptoms in some cultivars and was named “Planta Vinosa” (PV) due to the distinctive reddish color on stems and petioles, similar to that of red wine. The isolates were freeze-dried and maintained at −70 °C for several years. The inocula were recovered and multiplied through mechanical transmission with 0.05 M, pH 7.6 potassium phosphate buffer in soybean cv Don Mario 4800 and Davis. Soybean plants were grown under controlled conditions: 25 ± 2 °C and a 16:8 light–dark photoperiod (250 µmoL photon.m^−2^.s^−1^) and 65% humidity.

### 2.2. Biological Characterization

#### 2.2.1. Pathogenicity Test

Once multiplied, the four isolates were mechanically transmitted to a group of differential cultivars (Table 1), as suggested by several authors [11,32,33] The 10 inoculated plants per cultivar/isolate were maintained under greenhouse conditions (25 °C ± 2) until symptom development, at which point both local and systemic symptoms were recorded. Infection was confirmed 10 days post-inoculation (dpi) by analyzing the most recently fully expanded trifoliate leaf using the Plate-Trapped Antibody Enzyme-Linked Immunosorbent Assay (PTA-ELISA) [34]. Primary antibody was a rabbit polyclonal anti-soybean mosaic virus antiserum, produced in our lab (unpublished data), and the secondary antibody was a goat anti-rabbit IgG conjugated with alkaline phosphatase (BIO RAD, Hercules, CA, USA). The reaction was detected with the addition of 0.75 mg/mL of p-Nitrophenyl Phosphate, Disodium Salt (PNPP) (Agdia Inc., Elkhart, IN, USA), and quantified with the Synergy H1 microplate reader [BioTEK (Actually Agilent, Santa Clara, CA, USA)]. The plates were read every 15 min until OD = 1 of the positive control was reached.

#### 2.2.2. Aphid Transmission

Colonies of *Myzus persicae* Sulzer were bred on *Ipomea setosa* Nil. Two trials were performed, one using two aphids per plant and the other only one aphid per plant. For transmission, aphids were starved for 3 to 4 h, then allowed to feed on soybean plants infected with the different SMV isolates for a maximum period of one minute (acquisition); then they were allowed to feed on healthy soybean plants of cultivar Forrest (10 plants/isolate) for approximately 18 h. The plants were maintained under greenhouse conditions (25 °C ± 2) until the evaluation of transmission through visual symptoms and PTA-ELISA, following the previously described protocol.

#### 2.2.3. Seed Transmission

Twenty soybean plants of cultivar Forrest per studied isolate (MJ, M, NOA, and PV) were mechanically inoculated. The inoculated plants were maintained under greenhouse conditions (25 ± 2 °C) until maturity, when pods were harvested, and the percentage and degree of seed mottling were estimated. To evaluate seed transmission, all the harvested seeds were sown in individual pots, and seedlings were analyzed by PTA-ELISA using the first emerged trifoliate leaf.

### 2.3. Molecular Characterization

Total RNA was extracted from approximately 200 µg of the last fully expanded trifoliate leaf using the Trizol reagent method [35]. RNA was quantified using the nanodrop^®^ ND-1000 Spectrophotometer [NanoDrop Technologies, Inc. (Actually Thermo Fisher Scientific, Wilmington, DE, USA)].

Fragments corresponding to the CI and P1 genomic regions were amplified by RT-PCR, using the sets of primers described by Kim et al. [36] and Sherepitko et al. [37] (Table S1). RT-PCR was performed with the Access RT-PCR System (Promega Corporation, Madison, WI, USA), using as template 1 µg total RNA of the different isolates. RT-PCR conditions for the CI segment were as follows: cDNA synthesis at 48 °C for 45 min, 2 min at 94 °C, followed by 40 cycles of 30 s at 94 °C, 1 min at 60 °C, and 2 min at 68 °C, with a final extension of 7 min at 68 °C. To amplify the P1 fragments, thermocycling was programmed as follows: 48 °C for 45 min, 2 min at 94 °C, and 35 cycles of 30 s at 94 °C, 30 s at 55 °C, 1 min at 68 °C, and the last extension of 10 min at 68 °C.

Two pairs of primers, CP and NIb-CP (Table S1), were used for the amplification of the complete CP coding region. The RT-PCR for the CP segment was carried out with the same reaction mix and conditions as those used for P1. For the NIb-CP fragment Hot Start Taq Master Mix Kit (Qiagen GmbH, Hilden, Germany) was used, and the RT-PCR conditions were as follows: 15 min at 95 °C and 40 cycles of 30 s at 95 °C, 1 min at 53 °C, 1 min at 72 °C, and the last extension of 10 min at 72 °C. The amplified products were purified with the DNA cleaning and concentrator kit (Zymo Research, Irvine, CA, USA) and sequenced at the Genomic Unit of the Biotechnology Institute-INTA (Argentina). To reconstruct the full-length CP sequences, the amplicons generated with the CP and NIb-CP primers were assembled using the SeqMan tool (DNASTAR Inc., Madison, WI, USA). Identity analyses were performed using the NIb-CP segment, while phylogenetic inference was based solely on the CP region.

All sequences corresponding to each isolate were compared with one another, as well as with SMV isolates deposited in the National Center for Biotechnology Information database: NCBI—http://www.ncbi.nlm.nih.gov (accessed on 21 May 2022), using the Blastn algorithm, http://www.ncbi.nlm.nih.gov/BLAST (accessed on 22 May 2022) [38]. Sequence identities were analyzed with the LASERGENE 7.0 (DNASTAR Inc., Madison, WI, USA) program. Multiple alignments were performed with Clustal W, http://www.justbio.com (accessed on 22 May 2022). Maximum Likelihood (ML) phylogenetic trees were constructed with the MEGA X program employing the GTR + G as A best-fit model with 1000 bootstrap iterations [39]. RDP, GENECONV, MaxChi, BOOTSCAN, Chimaera, 3Seq, and SISCAN methods implemented in the RDP4 (Recombination Detection Program v.4.82) program [40] were used to detect recombination events between the different isolates under study. Only those events supported by at least four recombination detection methods and exhibiting a *p*-value > 0.01 were classified as positive.

### 2.4. Physiological Parameters

#### 2.4.1. Infection with SMV

Plants of *Glycine max* cv. Don Mario 4800 and Davis were inoculated with SMV isolates M, MJ, NOA, and PV. Symptomatic leaves were homogenized in 0.05 M potassium phosphate buffer (pH 7.6) to prepare the inoculum. Mechanical inoculation was performed at 7 days post-germination (dpg) by gently rubbing the first pair of unifoliate true leaves dusted with 600-mesh carborundum. At the time of inoculation, plants were at the VC (cotyledon) growth stage.

To evaluate systemically infected leaves, samples were consistently collected from the corresponding leaf position on different plants inoculated at the same time, specifically, the first trifoliate leaf at 4 days post-inoculation (dpi), prior to symptom expression (BS), and at 12 dpi, during late symptom expression (LS). For mock-infected controls, mechanical damage was induced using 600-mesh carborundum with 0.05 M potassium phosphate buffer (pH 7.6).

#### 2.4.2. Growth Parameters

A total of 12 plants per treatment were harvested at the end of the experiment (12 days dpi). To measure Fresh weight (FW) and Dry weight (DW), aboveground tissues were individually harvested. Dry Weight (DW) was assessed after oven-drying samples at 80 °C until a constant weight was reached. Leaf area was calculated from scanned images of plants at 4 and 12 dpi, using Image Pro Plus ver. 4.5.0.29 for Windows 98/NT/2000 image analysis software.

#### 2.4.3. Chlorophyll Fluorescence

Quantum efficiency of photosystem II (ΦPSII) photochemistry under ambient light conditions (250 µmoL photon m^−2^ s^−1^, 25 ± 2 °C) (ΦPSII) was measured using a pulse amplitude modulated fluorometer (FMS2, Hansatech Instruments, Pentney King’s Lynn, UK). Furthermore, leaves were dark-adapted using leaf clips for at least 30 min to allow full oxidation of the reaction centers (RC). Then, a 1 s actinic light pulse at an intensity of 3500 μmol photons m^−2^ s^−1^ was applied to induce maximum fluorescence emission, allowing for the measurement of the maximum quantum yield of primary photochemistry (Fv/Fm).

#### 2.4.4. Lipid Peroxidation

Lipid peroxidation levels [determined as thiobarbituric acid reactive substances (TBARS)] were measured in the first trifoliate leaf, according to Heath & Packer [41]. The samples were homogenized using a mortar and pestle under liquid nitrogen, thawed in 3% (*v*/*v*) trichloroacetic acid (TCA), and centrifuged at 13,000× *g*, 4 °C for 15 min. A fraction (100 µL) of the sample was mixed with 100 µL of 20% TCA + 0.5% thiobarbituric acid (TBA) and incubated at 90 °C for 20 min; then the samples were rapidly cooled on ice. The mixture was centrifuged at 13,000× *g* for 10 min. The supernatant was immediately analyzed using a spectrophotometer, with absorbance readings taken at 532 nm to detect malondialdehyde and at 600 nm to correct for background turbidity.

#### 2.4.5. Total Soluble Sugars and Starch

Extracts were obtained following Guan & Janes [42] 2 g of frozen tissue was ground in 2 mL buffer containing 50 mM HEPES-KOH (pH 8.3), 2 mM EDTA, 2 mM EGTA, 1 mM MgCl_2_, 1 mM MnCl_2_, and 2 mM dithiothreitol (DTT). The extracts were centrifuged at 15,000 rpm at 4 °C for 15 min, and the supernatant was used for soluble sugar determination. Soluble sugars were measured with anthrone reagent [43], using sucrose as a standard. Starch was determined in the pellet from reducing sugars released after hydrolysis with α amyloglucosidase [44], using glucose as a standard.

#### 2.4.6. Serological Virus Detection

Soluble proteins were extracted in coating buffer (Na_2_CO_3_/NaHCO_3_), pH 9.6, and quantified according to Bradford [45] without SDS. Samples were diluted to 5 μg total protein per well and detected by PTA-ELISA, with a polyclonal anti-SMV serum as described above. Bovine serum albumin was used as a standard for calibration curves. In all cases, six healthy samples and one SMV-positive sample per plate were used as controls. Reactions were quantified in the Thermo Labsystem MultisKan MS spectrophotometer, and samples were considered positive when OD_405_ was higher than the mean of healthy controls plus three times the standard deviation (cut-off) or 0.100. Finally, the relative virus concentration was calculated through the A405 of each sample/cut off.

#### 2.4.7. Statistical Analysis

The obtained data were subjected to a parametric analysis of variance (ANOVA), for which the assumptions of Normality and Homogeneity of variances for each variable used were tested. Significant differences (*p* < 0.05) between treatments were evaluated using a DGC multiple range test. All these analyses were carried out with the InfoStat program [46]. Statistical analyses were made between treatments, and the values were expressed relative to the control.

## 3. Results

The pathogenicity tests performed to characterize the four isolates under study did not allow us to group them with any of the strains previously described by other research groups. The phenotypic severity of the isolates showed differences, with the SMV-PV isolate being the most severe one, since it produced mosaic symptoms only in the susceptible cultivar Clark and caused symptoms of systemic necrosis in the other cultivars, except in Buffalo and PI 483084. On the other hand, the mildest isolate turned out to be NOA, which produced mosaic symptoms in Clark and Davis cultivars, and systemic necrotic symptoms only in the Kwanggyo cultivar (Table 1).

The percentages of transmission by aphids for each isolate, detected by PTA-ELISA, were proportionally similar in both trials (Table 2). The SMV-M, -MJ, and -NOA isolates had a similarly high percentage of aphid transmission (61–72%), whereas SMV-PV presented very low transmission capacity (12.5%). Seven days after transmission, all the infected plants presented symptoms, such as necrotic and chlorotic local lesions, chlorotic spots, and mosaic.

Seeds from cultivar Forrest derived from plants infected with isolate SMV-M presented the highest percentage of mottling (62%) and the highest rate of seed transmission (13%), whereas the seeds infected with the PV and MJ isolates were those that showed the lowest percentage and mottling severity (Table 3).

### 3.1. Phylogenetic Characterization and Recombination Analysis of the SMV Isolates

The complete sequences of the P1, CI, and CP gene regions of the isolates were deposited in the GenBank database (Table S2). The amino acid identity among isolates for each segment is presented in Table 4. CI and CP segments showed a great similarity (98.2%, 99.6%) in all the analyzed sequences, whereas for the P1 segment, the identity was significantly lower (69.2–69.8%) when the sequences of the M and MJ Isolates were compared with those of PV and NOA

**Figure 1:**
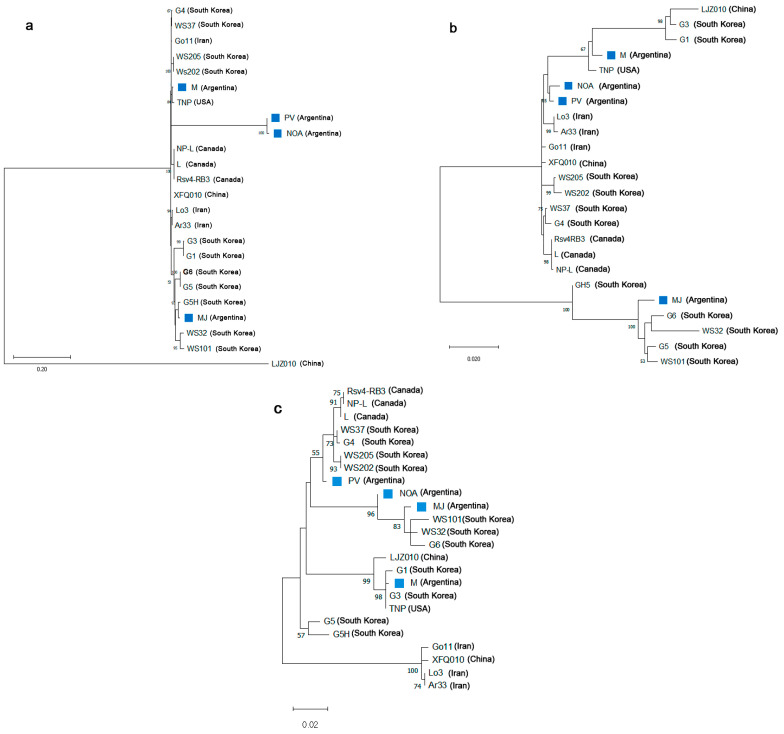
Phylogenetic trees constructed based on the nucleotide sequences of the P1 (**a**), CI (**b**), and CP (**c**) segments of Argentinean soybean mosaic virus isolates (MJ—Marcos Juarez, PV—Planta Vinosa, NOA, and M—Manfredi), together with other selected reference strains/isolates. Analyses were performed using the maximum likelihood method, with 1000 bootstrap replicates to assess the reliability of the groupings. Bootstrap values higher than 50% (1000 replicates) are indicated at the corresponding nodes. Abbreviations correspond to the names of the analyzed strains/isolates. GeneBank accessions of the soybean mosaic virus strains are as follows: TNP: HQ845735; Rsv4-RB3: JN416770; L: EU871724; NP-L: HQ166266; WS32: FJ640954; WS37: FJ640955; WS101: FJ640957; WS202: FJ640974; WS205: FJ640975; G1: FJ640977; G3: FJ640978; G4: FJ640979; G5: AY294044; G6: AF242845; G5H: FJ807701; Go11: KF135491; Lo3: KF135490; Ar33: KF297335; LJZ010: KP710866; XFQ010: KP710874.

The phylogenetic trees are presented in Figure 1a–c. The SMV-NOA and SMV-PV isolates clustered closely in the CP, P1, and CI regions, with bootstrap support values ranging from 55% to 100%, depending on the genomic segment analyzed. The SMV-M isolate grouped with the TNP strain (USA) in the analysis of the P1 fragment (bootstrap value 84%). For the CI region, it clustered with the TNP strain (52%) and with strains G1, G3, and LZ 010 (67%). In the CP region, the M isolate was closely related to G1, G3, and TNP, with strong bootstrap support (98%). Finally, in the CI and CP regions, the SMV-MJ isolate formed a strongly supported cluster with G5, G6, WS 323, and WS 101 (South Korea), with bootstrap values of 100% and 83%, respectively.

**Figure 2:**
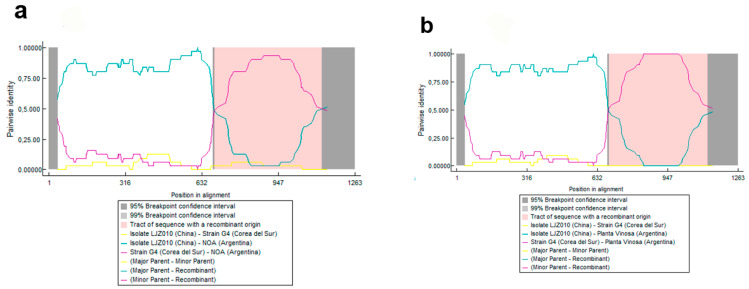
DNA Recombination events detected in the P1 segment of (**a**) NOA and (**b**) Planta Vinosa (PV) isolate with parental LJZ010 (light blue) and G4 (violet); the corresponding breakpoints at nucleotides 690 and 1128 for the NOA isolate and nucleotides 590 and 1185 for the PV isolate are included.

**Figure 3:**
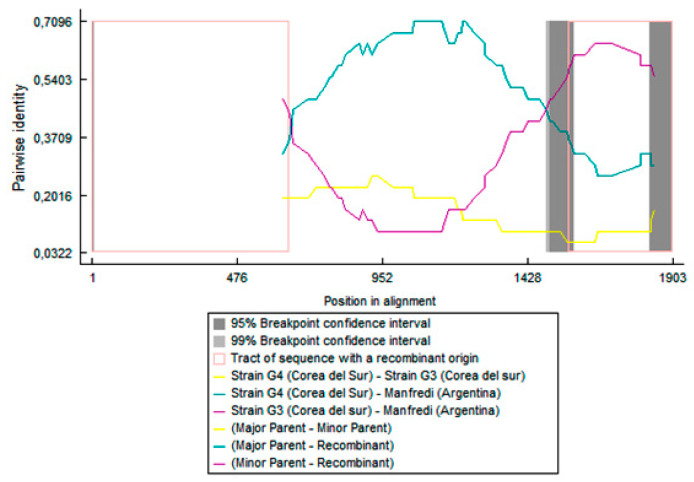
Recombination events detected in the CI segment of Manfredi (M) isolate with parental LJZ010 (light blue) and G4 (violet); the corresponding breakpoints at nucleotides 1560 and 1902 are included.

In the recombination analyses, all detected events were consistently predicted by multiple methods (RDP, GENECONV, MaxChi, BOOTSCAN, Chimaera, 3Seq, and SISCAN), with *p*-values below 0.05. These results suggest that the SMV-NOA, -PV, and -M isolates may have arisen by recombination (Figure 2 and Figure 3). The P1 segments of SMV-NOA and -PV isolates presented the same recombination event, with exchange points being nucleotides 690 and 1128 approximately for SMV-NOA and 690 and 1185 for SMV-PV. LJZ010 and G4 were detected as the major and minor parental sequences, respectively. On the other hand, in the analysis of the CI segment, SMV-M isolate, as the TNP strain, would be recombinant between G4 (parent major) and G3 (parent minor), and the exchange points for SMV-M were nucleotides 1560 and 1902. The analysis of the CP fragment showed no recombination events.

### 3.2. Early Physiological Alterations Caused by the Different Isolates of SMV

**Figure 4:**
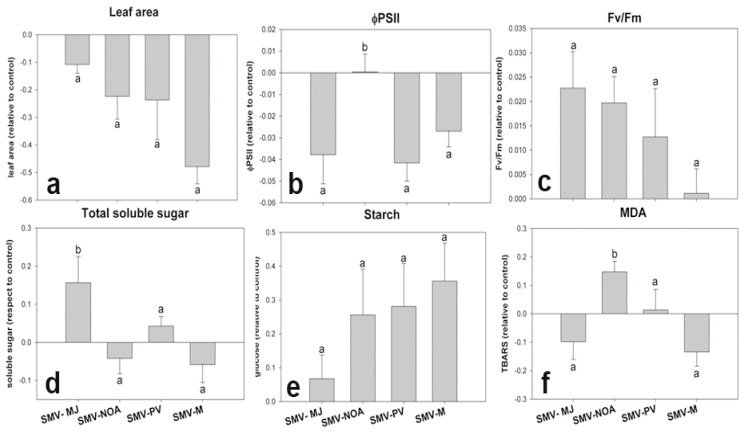
Early physiological alterations induced by different SMV isolates in soybean plants before macroscopic symptom appearance. (**a**). Leaf area; (**b**). quantum efficiency of photosystem—ΦPSII; (**c**). Fv/Fm; (**d**). total soluble sugars; (**e**). starch; (**f**). malondialdehyde—MDA. Sampling was carried out on the first trifoliate leaf 4 days after inoculation. Results are expressed as means ± SE of three independent experiments with at least three biological replicates each. Different letters indicate significant differences between treatments (DGC test. *p* < 0.05).

Before the development of viral symptoms (BS), SMV-NOA produced a differential behavior in quantum efficiency of photosystem II (ɸPII) and malondialdehyde (MDA) content in leaves with respect to the other isolates (Figure 4b,f). ɸPII can be used to estimate the photosynthetic performance, while MDA is a widely used marker of oxidative lipid injury. Likewise, SMV-MJ caused a significant increase in total soluble sugar content with respect to the other isolates (Figure 4d). All the SMV isolates exhibited a similar behavior in terms of maximum quantum yield of primary photochemistry (Fv/Fm), starch content, and leaf area (Figure 4a,c,e).

### 3.3. Physiological Alterations After Viral Symptom Development

In order to analyze the effect of infection by the different virus isolates over time, we measured Fv/Fm, starch content, and leaf area in the same leaf, eight days after the early measurements.

**Figure 5:**
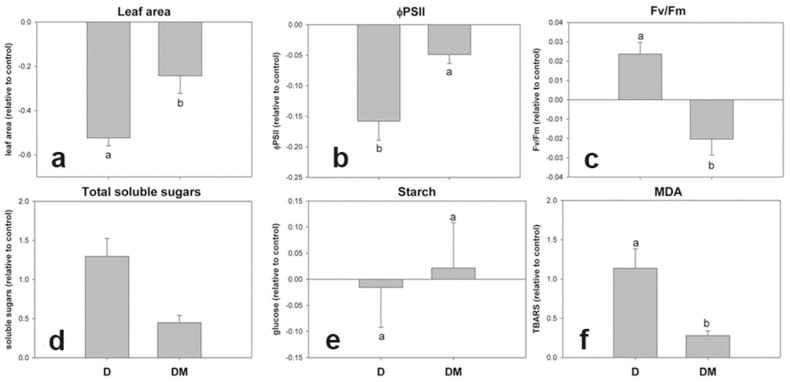
Physiological alterations induced by different isolates of SMV in soybean plants after the appearance of mosaic symptoms. (**a**). Leaf area; (**b**). quantum efficiency of photosystem—ΦPSII; (**c**). Fv/Fm; (**d**). total soluble sugars; (**e**). starch; (**f**). malondialdehyde—MDA. Sampling was carried out 12 days post-inoculation on the first trifoliate leaf. Results are expressed as means ± SE of three independent experiments with at least three biological replicates each. Different letters indicate significant differences between treatments (DGC test. *p* < 0.05).

**Figure 6:**
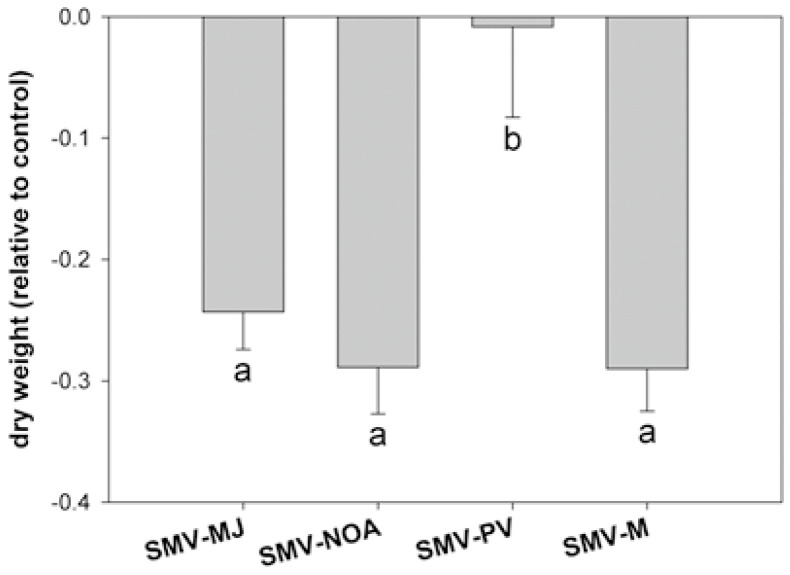
Dry weight of aboveground with respect to healthy control measured at 12 days post-inoculation—dpi (LS). Results are expressed as mean ± SE of three independent experiments with at least three biological replicates each. Different letters indicate significant differences between treatments (DGC test. *p* < 0.05).

SMV-NOA isolate caused an increase in MDA content, whereas (Figure 5f) SMV-M caused an increase in soluble sugar content, starch, and MDA, without changes in ΦPSII (Figure 5b,d–f). SMV-PV isolate did not affect biomass production (Figure 6). Moreover, relative viral concentration was measured after the appearance of symptoms, and no differences were observed among isolates (Figure S2).

**Figure 7:**
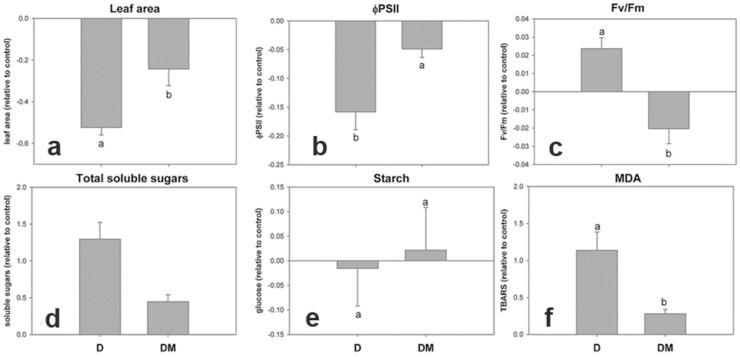
Physiological alterations induced by SMV in soybean plants of two susceptible cultivars after the appearance of mosaic symptoms. (**a**). Leaf area; (**b**). quantum efficiency of photosystem— ΦPSII; (**c**). Fv/Fm; (**d**). total soluble sugars; (**e**). starch; (**f**). malondialdehyde—MDA. The sampling was conducted 12 days after inoculation on the first trifoliate leaf. Results are expressed as ± SE of three independent experiments with at least three biological replicates each. Different letters indicate significant differences between treatments (DGC test. *p* < 0.05).

The SMV-MJ isolate was selected to study the response of two soybean cultivars susceptible to SMV infection, Don Mario 4800 and Davis. The soybean cv Davis showed more severe symptoms than Don Mario, not only when infected with the SMV-MJ isolate in this work. But also, when infected with the SMV-M isolate, which showed severe necrosis in the infected plants (unpublished data). Since the cultivar Davis showed lower infectivity than cultivar Don Mario, physiological measurements were taken after the macroscopic symptoms development (LS stage). Soybean cv Davis showed differential behavior with respect to Don Mario in leaf area, ΦPSII, Fv/Fm, and MDA (Figure 7).

## 4. Discussion

In this study, we performed the biological, molecular, and physiological characterization of four SMV isolates, three isolates obtained from different locations in the country (M, MJ, and NOA) and one that causes symptoms of severe necrosis in some cultivars (PV). Although the SMV-PV and SMV-NOA isolates shared a high degree of sequence identity in the analyzed regions (P1, CI, and CP) and exhibited the same recombination event that was predicted with a high level of confidence (detected by at least four programs with a *p*-value < 0.01), and their grouping in the phylogenetic trees suggest that they could belong to the same SMV strains, both differed in pathogenicity, percentage of seed mottling, percentage of transmission by seeds and aphids, and plant physiological response. The significant differences observed in pathogenicity and physiological parameters such as MDA content and ΦPSII may not be solely attributed to specific genotypic differences. Given that only partial viral genome regions were analyzed, it is possible that additional genomic regions, epigenetic factors, or specific host–virus interactions may contribute to the observed differences in pathogenicity. Therefore, further comprehensive genomic characterization will be required to better understand the molecular mechanisms underlying the phenotypic variation among isolates.

So far, four independent loci for resistance to SMV have been identified (*Rsv1*, *Rsv3*, *Rsv4*, and *Rsv5*). In addition, multiple resistance alleles were reported for the loci *Rsv1* and *Rsv3* [47,48]. The emergence of resistance-breaking isolates can be attributed to the use of resistant cultivars, with a limited base of resistance to SMV subjected to selection pressure, due to mutations and/or recombination of the different virus strains [20]. This may have been the case of SMV-PV isolate, since it produced a hypersensitive reaction in several cultivars, causing symptoms of necrosis in stem petioles and veins. In the pathogenicity tests, SMV-PV produced necrotic symptoms in all the evaluated cultivars, except for Buffalo and PI 483084, which contained *Rsv1-K-* and *Rsv1-h*-resistant genes. Proteins P3 and HC-Pro have been shown to be the effectors of resistance mediated by Rsv1. In addition, the amino acids 823, 953, and 1112 of P3 were found to be important for the induction of the lethal systemic hypersensitive response (LSRV) [49,50]. Thus, SMV-PV could have emerged as a consequence of mutations in P3 and/or HC-Pro cistrons, which broke the resistance conferred, at least, by the alleles *Rsv5*, *Rsv1*, *Rsv-1-t*, and *Rsv1-K*. Thus, efforts should be made to complete the sequencing of these two cistrons with the aim of understanding the biological differences between isolates, mainly considering that SMV-PV causes severe symptoms of necrosis.

The coat protein (CP) and helper component proteinase (HC-Pro) have been shown to play key roles in both aphid- and seed-mediated transmission of potyviruses, while the P1 cistron has also been identified as a determinant specifically influencing seed transmission [24]. A conserved amino acid motif, DAG, within the CP is commonly found across most potyviruses and is implicated in both transmission pathways. In the case of Soybean mosaic virus (SMV), HC-Pro appears to be involved in inducing seed coat mottling, likely through the partial suppression of Chalcone synthase (CHS) mRNA silencing [16,51].

In our study, we observed significant variation in transmission efficiency among the analyzed SMV isolates. The SMV-PV isolate exhibited the lowest rate of aphid transmission, while both SMV-PV and MJ isolates demonstrated reduced seed coat mottling, correlating with lower seed transmission efficiency. Notably, the DAG motif within the CP was conserved across all four isolates.

Furthermore, recombination events within the CP region are reportedly rare [52], a finding consistent with our results, as no recombination signals were detected in any of the four viral isolates using RDP analysis.

The P1 protein, first translated from the potyvirus genome, shows high sequence variability, which is thought to contribute to the virus’s ability to adapt to different hosts [53]. The analysis of the amino acid sequence of P1 showed that sequence identity was highest between the SMV-M and -MJ isolates and between SMV-PV and NOA isolates. The SMV-M isolate is the one that causes the highest percentage and severity of seed mottling, as well as the highest percentage of transmission by seeds and physiological differences at the LS stage. It has been shown that SMV P1 protein interacts strongly with the Rieske Fe/S protein of soybean cytochrome b6f [54], an essential component of the chloroplast electron transport chain. The interaction between the chloroplast and the invading viruses is believed to play a critical role in viral infection and pathogenesis [55]. In this context, our results, together with previous findings from our group [29], suggest that the observed decrease in ɸPSII may reflect a disruption of the photosynthetic electron transport chain, likely leading to a reduction in CO_2_ fixation, particularly in plants infected with the SMV-MJ and PV isolates. Chloroplasts are the main source of intracellular reactive oxygen species (ROS) generation in green tissues, mainly under stress conditions. The virus’s ability to impair chloroplast function and disrupt the photosynthetic electron transport chain ultimately leads not only to a decrease in the carboxylation activity but also to ROS increase [30,56,57]. Our results showed an increase in MDA content in soybean leaves inoculated with SMV-NOA and SMV-M isolates at the LS stage. MDA is a marker of oxidative lipid damage caused by stress [58]. Notably, SMV-NOA was the only isolate that produced an early increase in MDA content, without alteration of ɸPSII. Although this does not constitute direct evidence of oxidative damage as an early marker, it suggests that lipid peroxidation may occur prior to detectable impairment of chloroplast function in SMV-NOA-infected plants. We therefore hypothesize that early MDA accumulation may reflect an initial oxidative imbalance that occurs independently of photosynthetic decline. Further studies will be required to confirm whether MDA accumulation can serve as an early indicator of SMV-induced oxidative stress and to determine whether this response is associated with viral genomic features outside the region analyzed, given the high sequence identity between SMV-NOA and SMV-PV. Likewise, since SMV-NOA and -PV had high nucleotide sequence identity in the analyzed fragment, we suggest that the physiological response at the chloroplast level could be related to other sequences that should be further explored.

Chloroplast alteration during SMV infection might be directly related to soluble sugar production. Present and previous results of our group have shown an increase in soluble sugars with SMV-MJ infection [30]. It is possible that the increase in soluble sugars observed in the compatible interaction between soybean and all SMV isolates is associated with the recycling of cellular components resulting from chloroplast damage. The accumulation of soluble sugars and the decrease in the ΦPSII and Fv/Fm suggest that the increase could be related to a greater import or lower export of sugars, or from intracellular recycling [29,56]. These possible sugar sources are not mutually exclusive but might be operating in combination and must be explored in future studies.

SMV-MJ, one of the most severe isolates in terms of the reactions it produces in differential cultivars, showed early sugar alteration. On the other hand, the differential behavior of that virus isolate was found to occur not only between cultivars with different resistance [59] or between susceptible and resistant cultivars [58], but also between susceptible cultivars. The soybean cv Davis showed more severe symptoms than Don Mario under infection with both SMV-MJ and SMV-M isolates (unpublished data), suggesting that host genotype may influence symptom severity even among susceptible cultivars. However, the underlying causes of this differential response remain to be elucidated. Likewise, mechanical transmission had low efficiency in the SMV-M, SMV-NOA, and SMV-PV isolates, suggesting a differential interaction of the same viral genome with different susceptible host plant genomes. In conclusion, knowing the physiological bases of viral infections and the mechanisms underlying plant infection by different races of viruses will contribute to the development of plants with tolerance to viral diseases.

## Figures and Tables

**Table 1:** Reactions of soybean differential varieties to four soybean mosaic isolates from Argentina.

Cultivar	Genotype	G1	G2	G3	G4	G5	G6	G7	MJ	M	NOA	PV
I												
Clark	rsv	-/M	-/M	-/M	-/M	-/MN	-/M	-/M	N/M	N/M	N/M	N/M
Davis	rsv	-/-	-/-	-/-	N/MN	-/M	-/M	-/M	N/MN	N/-	N/M	N/MN
York	Rsv-5	-/-	-/-	-/-	N/MN	-/MN	-/M	-/M	N/MN	N/N	-/-	N/MN
Ogden	Rsv-1t	-/-	-/-	N/N	-/-	-/-	-/-	N/N	-/-	N/MN	-/-	-/N
Kwanggyo	Rsv-1K	-/-	-/-	-/-	-/-	N/N	N/N	N/N	N/N	-/-	N/N	N/MN
Buffalo	Rsv-1b	-/-	-/-	-/-	-/-	-/-	-/-	N/N	-/-	N/-	N/-	N/-
II		MS1	MS2	MS3								
Clark	rsv	M	M	M					N/M	N/M	N/M	N/M
Davis	rsv	-	-	M					N/MN	N/-	N/M	N/MN
York	Rsv-5	M	M	M					N/MN	N/N	-/-	N/MN
Ogden	Rsv-1t	-	M N	M N					-/-	N/NM	-/-	-/N
Kwanggyo	Rsv-1K	-	M LL	M					N/N	-/-	N/N	N/MN
Buffalo	Rsv-1b	-	-	N					-/-	N/-	N/-	N/-
III		A	B	C	D	E						
Clark	rsv	M	M	M	M	M			M	M	M	M
Davis	rsv			M	M	N			N	-	M	N
York	Rsv-5			M	M	N			N	N	-	N
Ogden	Rsv-1t	N	N	N	N					N	-	N
Kwanggyo	Rsv-1K			M	M	N			N	-	N	N
Buffalo	Rsv-1b			N	N					-	-	-
PI 96983	Rsv-1t				N				N	-	-	N
PI 483084	Rsv-1h					N				-	-	-

References: Differential cultivars described by I Cho & Goodman [11], II Almeida [32], III Shigemori [33], M = mosaic, N = necrosis, LL = local lesion, S = symptoms on non-inoculated primary leaves, ( ) = indiscernible, -/- = reaction on inoculated primary leaves/reactions on non-inoculated trifoliate leaves.

**Table 2:** Transmissibility (%) of soybean mosaic virus Argentinian isolates by *Myzus persicae*.

	M	NOA	MJ	PV
2 aphids per plant	72	67	61	12.5
1 aphid per plant	37.5	38	20	10

**Table 3:** Seed transmission and mottling of different soybean mosaic virus isolates in Forrest cultivar.

Isolate	% of Not Spotted Seeds	% Spotted Seeds	Mottling Severity *	% Seed Transmission (PTA-ELISA)
Planta Vinosa	65	35	1-2	7
Marcos Juarez	58	35	1-2	7
NOA	60	39	1-3	10
Manfredi	38	62	1-4	13

* Mottling severity 1: mottling covers less than 20% of the seed surface; 2: mottling covers between 20 and 40% of the seed surface; 3: mottling covers between 40 and 60% of the seed surface; 4: mottling covers more than 60% of the seed surface.

**Table 4:** Percentages of amino acid sequence identity between the four soybean mosaic virus isolates.

Segment	Isolates	Similarity (%)			Divergence (%)		
		MJ	NOA	PV	MJ	NOA	PV
**P1**	M	95.9	**53.1**	**53.6**	4.2	31.4	30.6
	MJ	-	**51.1**	**51.4**	-	32.3	31.9
	NOA	-	-	99.1	-	-	0.9
**CI**	M	90.2	97.8	97.7	10.8	2.2	2.3
	MJ	-	91	91	-	9.8	9.8
	NOA	-	-	99.5	-	-	0.6
**CP**	M	92.8	94	95.2	7.8	6.4	5
	MJ	-	96.7	94.2	-	3.4	6
	NOA	-	-	97.5	-	-	2.5

## Data Availability

All the data is available with the corresponding authors, and it will be made available on request.

## Supplementary Materials

The following supporting information can be downloaded at https://www.mdpi.com/article/10.3390/v17070995/s1, Figure S1: Locations of soybean fields from which soybean mosaic virus isolates were collected. Table S1: Primers used to amplify the CP, P1, and CI genomic regions. Table S2: GenBank accession numbers of three regions of soybean mosaic virus genome, corresponding to four different Argentinian isolates. Figure S2: Comparison of the relative virus concentrations among the four SMV isolates, determined by PTA-ELISA absorbance readings at 405 nm (OD_405_).
