# Supplementary material for: Biological, Molecular, and Physiological Characterization of Four Soybean Mosaic Virus Isolates Present in Argentine Soybean Crops
Source: Viruses. 2025 Jul 16;17(7):995. doi: 10.3390/v17070995 (PMC12298932; doi:10.3390/v17070995)
Supplement: Supplementary file 1 [file viruses-17-00995-s001.zip › Table S1.pdf]

Supplementary file 1: Primers used to amplify the CP, P1 and CI genomic regions

Genomic region	Primer	Primer sequence	Fragmet size
CP*	SMV-CPf<br>SMV-CPr	5'-CAAGCAGCAAAGATGTAAATG-3'<br>5'-GTCCATATCTAGGCATATACG-3'	469 pb
P1	SMV-P1f<br>SMV-P1r	5'-AGTCAAATGGCAACAATCATG-3'<br>5'-GGGAGTAGTGCTGAATATCC-3'	934 pb
CI	SMV CI5'<br>SMV CI3'	5'-GCATTCAACTGTGCGCTTAAAGAAT-3'<br>5'-TTGAGCTGCAAAAATTTACTCACTT-3'	1385 pb
Nib-CP*	Nib-CPF<br>Nib-CPR1	5'-AGCAAAGAGCTTATGCATC-3'<br>5'- CCTTCAACCATTGGAAGATTCA-3	500

*The PCR products of primer sets 'CP and NIb-CP overlap and the obtained sequences were combined to generate the full sequences of the CP genes
