# Supplementary material for: Biological, Molecular, and Physiological Characterization of Four Soybean Mosaic Virus Isolates Present in Argentine Soybean Crops
Source: Viruses. 2025 Jul 16;17(7):995. doi: 10.3390/v17070995 (PMC12298932; doi:10.3390/v17070995)
Supplement: Supplementary file 1 [file viruses-17-00995-s001.zip › Table S2.pdf]

Supplementary file 2: GenBank Accession numbers of three regions of Soybean mosaic virus genome, corresponding to four different Argentinian isolates.

Soybean mosaic virus region	Isolate	GenBank Accession number
P1	M	MH746624
P1	MJ	MH763836
P1	NOA	MH795799
P1	PV	MH785076
CI	M	MH672689
CI	MJ	MH683726.
CI	NOA	MH678613.
CI	PV	MH688059
NIb-CP	M	MW187865
NIb-CP	MJ	MW187866
NIb-CP	NOA	MW187867
NIb-CP	PV	MW187868
