# Supplementary figures and images for: Biological, Molecular, and Physiological Characterization of Four Soybean Mosaic Virus Isolates Present in Argentine Soybean Crops
Source: Viruses. 2025 Jul 16;17(7):995. doi: 10.3390/v17070995 (PMC12298932; doi:10.3390/v17070995)

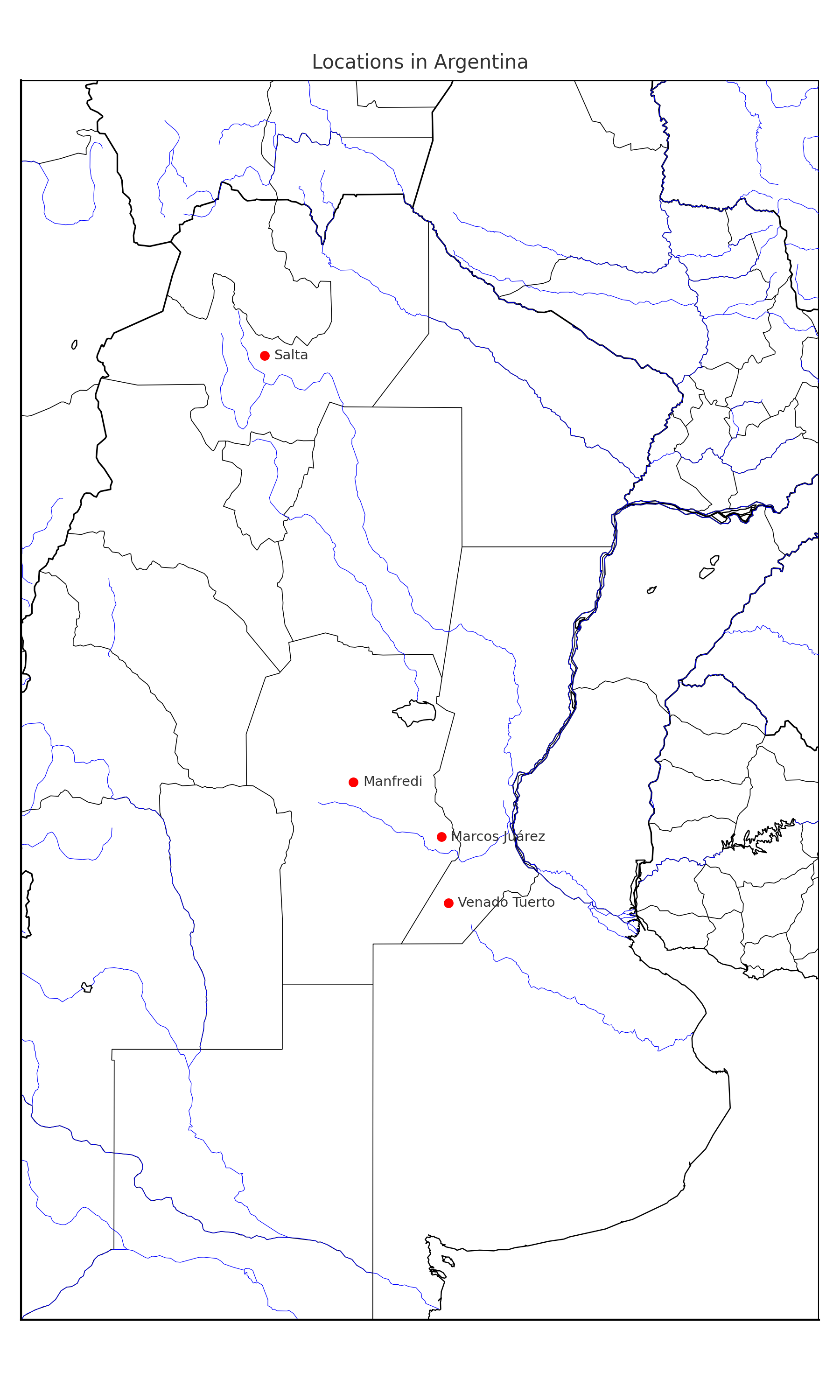

Supplement: Supplementary file 1 [file viruses-17-00995-s001.zip › Figure S1.tiff]
